# Does the mouse tail vein injection method provide a good model of lung cancer?

**DOI:** 10.12688/f1000research.17964.1

**Published:** 2019-02-15

**Authors:** Nensi Shrestha, Zabeen Lateef, Orleans Martey, Abigail R. Bland, Mhairi Nimick, Rhonda Rosengren, John C. Ashton

**Affiliations:** 1 Department of Pharmacology and Toxicology, University of Otago, Dunedin, 9054, New Zealand

**Keywords:** human cells, lung cancer, mouse model, tail vein

## Abstract

Lung cancer drug development requires screening in animal models. We aimed to develop orthotopic models of human non-small lung cancer using A549 and H3122 cells delivered by tail vein injection. This procedure has been used previously for a mouse lung cancer (Lewis lung carcinoma) and as a model of human breast cancer metastasis to lung. We report that the procedure led to poor animal condition 7-8 weeks after injection, and produced lesions in the lungs visible at necropsy but we were unable identify individual cancer cells using immunohistochemistry. We conclude that if this method is to produce a model that can be used in drug experiments, improvements are required for cancer cell detection post mortem, such as by using of a fluorescently tagged human lung cancer cell line.

## Introduction

Lung cancer causes more deaths worldwide than any other cancer in both males and females ^1^. Good animal models of lung cancer are essential if treatments are to improve, but there are disadvantages to existing models of lung cancer. Non-small cell lung cancer (NSCLC) comprises approximately 85% of all lung cancer ^2^, and therefore represents the lung cancer subtype where good models are most needed.

Mouse models of lung cancer fall into several categories. The first division that can be made is whether the cancer cells are of mouse or human origin. Cancer cells of mouse origin may be grafted onto a host mouse, or induced in tissues by genetic modification, chemical means, or spontaneously. Neither fully recapitulates human cancer. To study the effects of a drug on human cancer in mouse models requires cancer cell xenografts in immunocompromised mice, such as nude or severely immune comprised (SCID) mice. This may be as a solid tumour in the mouse flank or for increased pharmacokinetic validity, an orthotopic model where cells are directly grafted into the lungs. Grafting cancer cells into mouse lungs may take place by several different methods. The cells may be directly injected into the lung, (i.e., by intrathoracic implantation via puncture ^3^), or the cells may be introduced into the airways of the mouse, causing a bronchial tumour ^4^.

An advantage of intrathoracic implantation, by direct puncturing through the intercostal space to lung parenchyma, is that it avoids thoracotomy or intubation, but the method is disadvantaged by the risk of pneumothorax, intrathoracic haemorrhage and haemoptysis ^3^. Methods of bronchial implantation without surgical thoracotomy have been developed ^4^, but these are disadvantaged by a risk of death during cancer cell inoculation. We therefore experimented with another method of cancer cell inoculation in the mouse lung; engraftment via vascular delivery and pulmonary entrapment. This method has been successfully used to create lung tumour nodules in the lungs of immunocompetent mice using Lewis lung carcinoma cells ^5^, but to our knowledge has not been used to study lung cancer using human lung cancer cells in immunocompromised mice. We therefore carried out a study using SCID mice, inoculating them via tail vein injection either with A549 or H3122 human adenocarcinoma cells. A549 cells are sensitive to Kirsten sarcoma virus protein (Kras) inhibition ^6^ and the H3122 cells are sensitive to anaplastic lymphoma kinase (ALK) inhibition ^7^.

## Methods

### Cell culture

Human lung adenocarcinoma cells (A549) were maintained in complete growth media (RPMI1640, ThermoFisher, USA) with 2% heat-inactivated foetal bovine serum (FBS, Sigma-Aldrich, NZ). Human NSCLC adenocarcinoma cells harbouring the EML4-ALK variant one (H3122) were maintained in 5% FBS RPMI 1640. All cell lines were also maintained in 2 mM L-glutamine and 1% streptomycin/penicillin (100 μg/mL, Sigma- Aldrich, AU), and grown in a humidified incubator at 5% CO _2_, 95% O _2_ and 37°C. At 80–90% confluence, cells were passaged with 1X TrypLE (A459 and H3122 cell lines, ThermoFisher, NZ).

### Animal housing and care

Male immunocompromised SCID mice were purchased from Animal Resources Centre, Australia. All animal experiments were performed after approval by the University of Otago (AEC #9/17). SCID mice were housed in pathogen-free condition with sterile woodchip bedding supplied with sterile food (Reliance rodent diet, Dunedin, NZ) and water. Mice were kept in a room maintained at temperature of 21–24°C on a scheduled 12 h light/dark cycle.

### Tail vein injection

A total of 48 male 12-week-old SCID mice, 20–30 g in weight, were divided into two identical experiments comprised of 24 mice each. As this is a method development study, no data existed with which to carry out a power analysis. Instead, we designed our experiment based on the maximum number of SCID mice we could import in one batch, dividing the mice into two groups in order to trial two different human adenocarcinoma cell lines. Mice were then sub-divided equally between 3-, 5-, and 8-week duration experiments. Two mice were not injected with cells. As this was not a hypothesis test study, we did not allocate mice to groups according to a randomization protocol, but assigned mice to groups sequentially by individual cage that they were housed in (allocated independently by animal technicians). In each experiment, mice were restrained and injected into the medial tail vein ( Figure 2) with either 1x10 ^5^ A549 cells or 1x10 ^5^ H3122 cells suspended in 100 µl of phosphate buffer saline (PBS). Mice were weighed daily and monitored for mobility, respiratory distress, and signs of pain daily for up to 8 weeks. A weight loss of more than 20% was deemed to be unacceptable and would lead to early sacrifice of the mouse. At the end of 3 weeks in each experiment 4 mice from each group (i.e., 8 mice across the two repeats of the experiment) were euthanized by CO _2_ exposure and perfused with isotonic PBS followed by 10% formalin. Organs were rapidly removed, weighed and kept in 10% formalin for 48 h followed by 30% starch solution for 24 h at 4°C. The organs were embedded in Optimal cutting temperature compound (OCT) and snap-frozen in liquid nitrogen. Organs removed in this way were brain, kidney, spleen, liver, lungs, heart, and testes. Lungs were quickly photographed prior to freezing and fixation. This was repeated for another 4 mice in each group at 5 weeks and 7–8 weeks following injection, and compared with lungs taken from SCID mice that were not injected with lung cancer cells, from another experiment.

**Figure 1.**
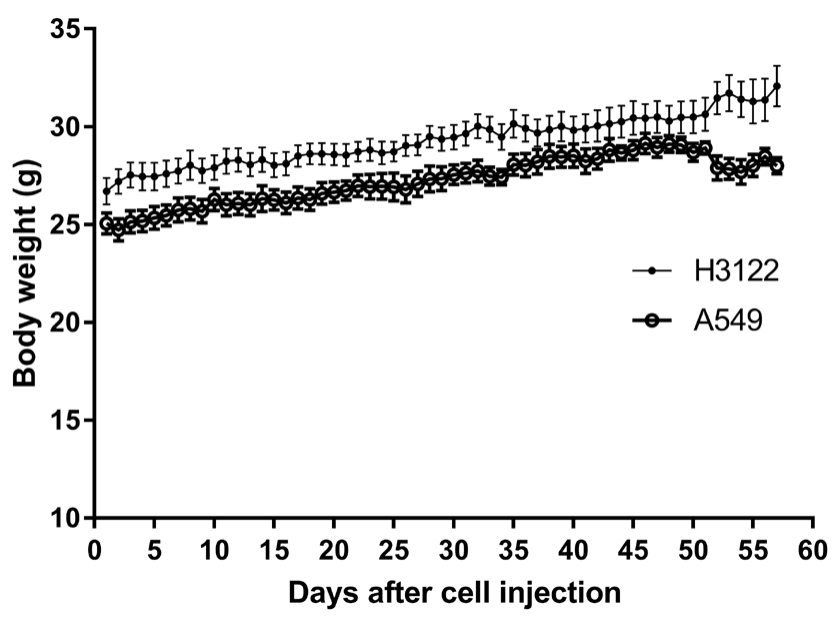
Body weight of SCID mice injected with A549 or H3122 human lung adenocarcinoma cells via the tail vein over 8 weeks. Data points are means and error bars are SEM. Up to week three there were fur mice for each cell type. Four mice were then sacrificed, and another four mice at week 5.

**Figure 2.**
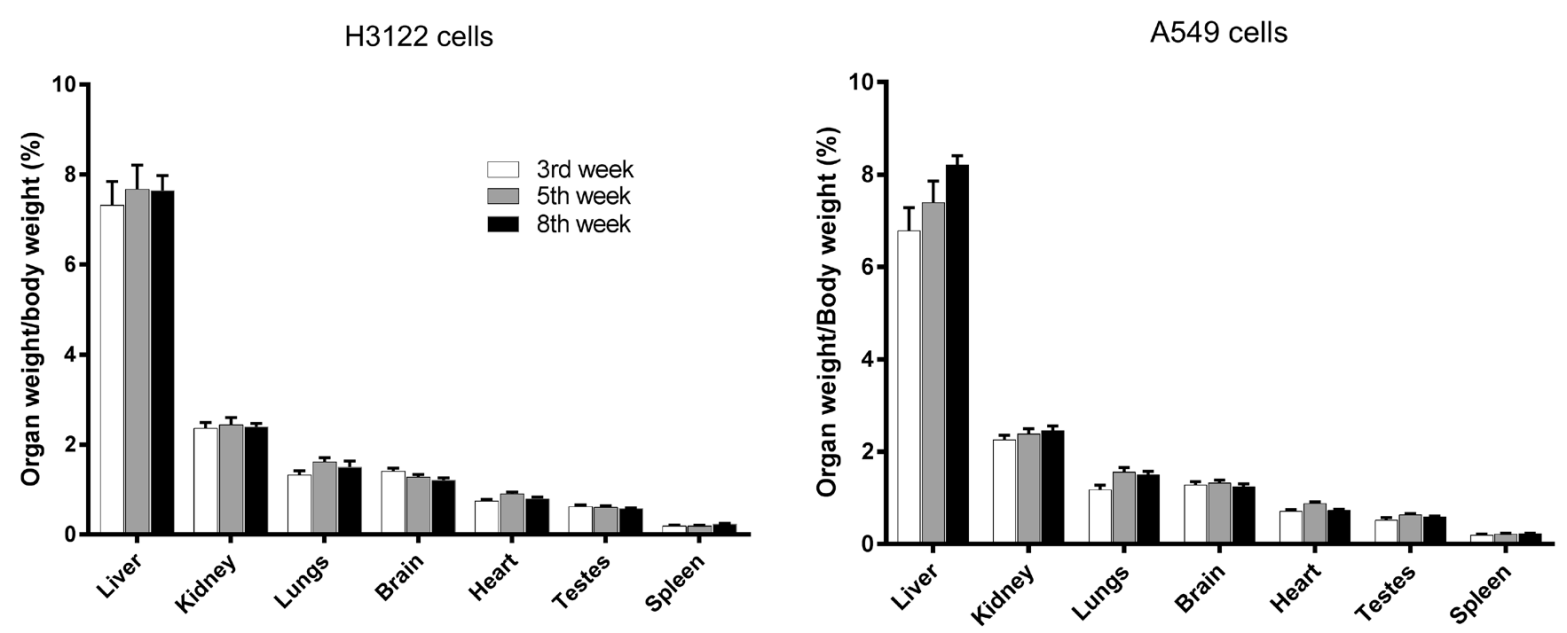
Organ weights of SCID mice injected with A549 or H3122 human lung adenocarcinoma cells via the tail vein at 3, 5, and 8 weeks after injection. Bar heights are means and error bars are SEM (n=4).

### Histology and immunohistochemistry

Frozen, fixed lungs were embedded in OCT and 6-µm sections cut and mounted on poly-L-lysine-treated frosted slides. Mounted sections were either stained with haematoxylin QS (H-3404, Vector laboratories, USA) and eosin (Sigma Aldrich, USA) or probed with antibodies for ALK (Cat# 3633, RRID:AB_11127207, Cell Signaling Technology, USA phospho-ALK (Cat# 6962, RRID:AB_10828357, Cell Signaling Technology, USA) (H3122 cell injected mice only) or human Ki67 Cat# M7249, RRID:AB_2250503, (Dako, Denmark and Abcam, UK).

Lung sections were fixed with acetone and methanol (1:2) solution for 10 min and were incubated with haematoxylin followed by eosin. Excess of eosin was removed by rinsing in 95% ethanol and slides were dehydrated in a series of 95%–100% ethanol. The sections were then soaked in xylene and mountant in DPX mounting solution.

For immunohistochemistry, lung sections were fixed with acetone for 10 min at room temperature and then treated with 0.3% of hydrogen peroxide in methanol for 20 min. Prior to incubation with antibodies, antigen retrieval was performed by boiling at 95°C in either water bath or in a decloaker chamber (Biocare Medical, USA) in citrate buffer (10 mM citric acid, 0.05% Tween-20) pH 6 for 20–30 min (Ki67) and EDTA buffer (1 mM EDTA and 0.05% Tween 20) pH 9 for 30–40 min (ALK/p-ALK). A range of blocking techniques were also trialled, including avidin-biotin blocking for biotinylated secondary antibodies. After incubation with primary antibodies overnight, washed slides were then incubated for up to 2 h with either goat anti-Rabbit IgG, HRP conjugate (Cat# 401353-2ML, RRID:AB_10690659, Millipore, US) or for up to 45 min with biotinylated goat anti-rabbit IgG (Cat# 550338, RRID:AB_393618BD, Biosciences, USA) for subsequent labelling with HRP-conjugated streptavidin( Cat# PA5-54066, RRID:AB_2639134, Thermo Fisher Scientific, USA). Following washing, all slides with stained with 3,3'-diaminobenzidine (DAB,BD Pharmingen, USA). Coverslip mounted sections were then examined by two examiners blinded to the treatment groups using a Nikon RM229 microscope.

### Statistical analysis

Weight gain over time was analysed using linear regression. Organ weights at necropsy were analysed using one-way ANOVA with Bonferroni post hoc tests. All statistical analyses were carried out using GraphPad Prism v7.

## Results

### Mouse weight

Mice that had been inoculated with A549 human lung cancer cells were slightly heavier than mice inoculated with H3122 cells, as shown by a 1.8 g difference in weight at baseline ( Figure 1; F = 884.5. DFn = 1, DFd = 111, P<0.0001, linear regression). However, this gap was maintained during the experiment, such that mice inoculated with A549 cells showed no difference in rate of weight gain from those inoculated with H3122 cells (0.068 g/day and 0.075g/day, respectively, a non-significant difference, F = 2.549. DFn = 1, DFd = 110, P=0.1133, linear regression). However, at over 50 days after inoculation, mice began to show signs of distress, manifested by hunched posture, immobility, rough coats, and laboured breathing. One mouse had proptosis (protruding eyes); this mouse was sacrificed, and all other mice were then sacrificed within a day (i.e., mice in the 8-week group were sacrificed in the 8 ^th^ week after injection). At this point, mice inoculated with A549 cells appeared to lose weight by 0.08 g/day, although this was not significant (F = 1.52. DFn = 1, DFd = 6, P=0.2638, linear regression). Raw values for body weight, alongside all other raw data, are available on figshare ^12^.

### Organ weight

**Figure 3.**
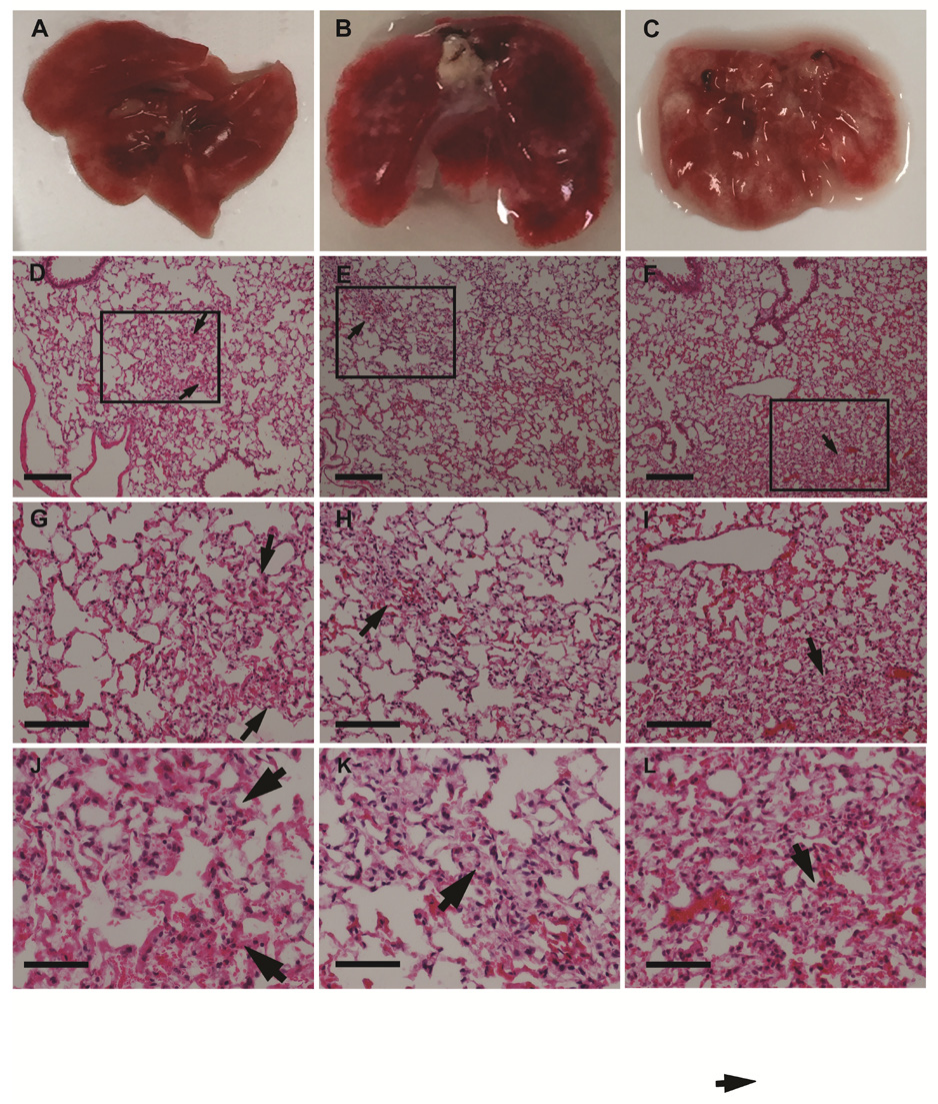
Gross appearance and histology of the lungs of SCID mice injected with H3122 human lung adenocarcinoma cells via the tail vein. (A-C) lungs from mice injected with cells at 3 (A), 5 (B), and 8 (C) weeks after cell injection. Note the white patches on the lungs, which appeared by week 5; D-F. Haematoxylin and Eosin stain of lungs from mice at 3 (D), 5 (E), and 8 (F) weeks after cell injection using a 10x objective. Squares and arrows indicate areas of high cell density; (G-I) Area shown in insets from panels (D-F) respectively, using a 20x objective. (J-L) Area shown in insets from images (D-F), respectively, using a 40x objective. Scale bars: D-I 100 µm; J-L 50 µm.

**Figure 4.**
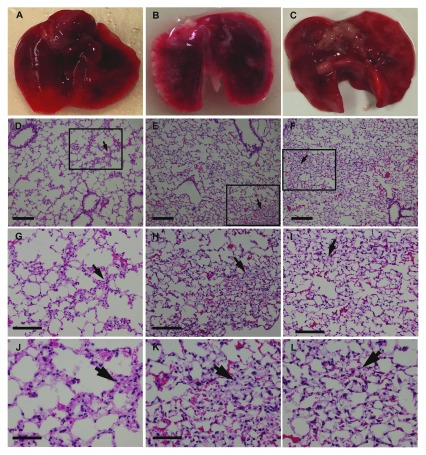
Gross appearance and histology of the lungs of SCID mice injected with A549 human lung adenocarcinoma cells via the tail vein. (A) lungs from a mouse that did not receive a cancer cell injection; B-C. lungs from mice injected with cells at 5 (B), and 8 (C). Note the white patches on the lungs, which appeared by week 5 (as for H3122 cells above). (D-F) Haematoxylin and Eosin stain of lungs from mice at 3 (D), 5 (E), and 8 (F) weeks after cell injection using a 10x objective. Squares and arrows indicate areas of high cell density. (G-I) Area shown in insets from images D-F respectively, using a 20x objective. (J-L) Area shown in insets from images (D-F) using a 40x objective. Scale bars: D-I 100 µm; J-L 50 µm.

At necropsy, organs were weighed; there were no significant (P > 0.05, one-way ANOVA with Bonferroni post hoc tests) differences between organ (including lung) weights taken at weeks 3, 5, or 8. ( Figure 2). However, examination of the lungs showed that numerous superficial white opacities began to appear by week 5, minimally apparent in week 3 mice lungs and absent in a control mouse (no cancer cells injected) ( Figure 3A-C and Figure 4A-C).

### Histological and immunochemical staining

Haematoxylin and eosin staining of the lungs did not show tumour cell nodules with distinct edges ( Figure 3 and Figure 4) consistent with the dispersed cancer cell pattern observed by Rashid et al. ^8^ using breast cancer cells. Notably, in earlier weeks for both lung cancer cell types, lung sections consisted of a sparse network of bronchioles, and alveolar ducts and sacs with infrequent areas where cells were aggregated in the parenchyma ( Figure 3– Figure 4). These areas were more extensive by week 5, and increased through to week 8.

However, we were unable to confirm that areas of cellular density corresponded to cancer cells, as we were unable to obtain positive staining for tumour cell markers using immunohistochemistry. In mice inoculated with ALK-positive H3122 cells, we did not find specific staining with either ALK or p-ALK antibodies (with secondary antibody only sections showing high amounts of non-specific staining). Similarly, when we looked in lung sections from mice inoculated with either cell, we could not distinguish primary antibody specific from non-specific labelling for any human cell marker, including Ki67. Although we used a range of antigen-retrieval techniques and different secondary antibodies (both directly conjugated to HRP, and biotinylated) we could not detect any primary antibody specific labelling in inoculated mouse lungs compare to control mouse lungs. There are at least two possible reasons for this. First, cancer cells may have been present but not detected by immunolabelling methods sufficiently due to low concentrations of secondary antibody. This may be because of over-fixation with formalin. To test this hypothesis will require a repeat of the experiment, testing a range of fixation methods or other visualisation methods (such as fluorescently labelling cells). As SCID mice are only available by importation in New Zealand, the country in which these experiments were carried out, this was not possible in the current study. But it is also possible that cancer cells have failed to engraft in lung parenchyma, such that cellular aggregations in haematoxylin and eosin-stained sections were either artefacts or else pathological features secondary to embolism. This second interpretation, however, is difficult to reconcile with the time-dependent appearance of superficial lesions on the lungs, loss of body condition, and increasing density of cells in lung histology, most apparent 8 weeks after tumour cell injection.

## Discussion

Lung cancer drug development is a difficult process, and using mouse models to screen novel drugs is a critical part of it. Development of new models is therefore part of the process of lung cancer drug development. We carried out these experiments in order to test whether tail vein injection of two commonly used human lung adenocarcinoma cell lines could recapitulate aspects of human lung cancer, to facilitate efficient drug testing. This procedure has successfully been used previously only with one mouse lung cancer cell line (Lewis lung carcinoma) ^5^, where female C57 immunocompetent mice, age 6 weeks, were tail vein injected with 2 × 10 ^6^ cells in 100 µl PBS). However, although our procedure did lead to poor animal condition in SCID mice 7–8 weeks after injection, as well as lesions in the lungs apparent at necropsy and histological differences, we were unable to identify individual cancer cells using immunohistochemistry. However, we do not yet conclude because of this that a useful model of lung cancer may not be produced by this method.

The pathological consequences of the tumour cell injection are consistent with thromboembolism, with areas of apparent hypoxia on the lungs at necropsy. In other types of cancer, tail vein injection has tended to lead to sudden death due to a thromboembolism. This and the lack of a more gradual progression of tumour burden has led to criticism of tail vein injection as a model of breast cancer metastasis ^8^. Cancer cell injection into any blood vessel is potentially embolic ^9^, and thromboembolism is a significant cause of death in human lung cancer ^10^, subsequent to the development of tumour nodules. Thus, due to the lack of visible lung cancer nodules using the tail vein injection method, the sudden decline in the mice (at 7–8 weeks after injection), the signs of ischaemia in mouse lungs beginning at week 5, but the difficulty in detecting individual cancers in our experiments, we conclude that the model requires further development if it is to be of value in drug development. We outline a strategy for this in our concluding paragraph below.

First, confirmation of hypoxic lesions in the lungs could be ascertained by tetrazolium chloride staining in fresh lung slices ^11^. Second, the structure of the lung is such that relatively strong fixation methods were required to ensure good morphology—more so than for most other tissues in our experience—which can compromise antigen exposure. We aim to overcome this problem in a future experiment through use of a fluorescently-tagged human lung cancer cell line. This approach will facilitate detection of individual cancer cells (by fluorescence microscopy or flow cytometry). Notably, in the study referenced above, Lewis lung carcinoma cells were labelled with GFP, and were thereby visualised by fluorescence microscopy in dissected lung tissue ^5^.

## Data availability

Raw data on body and organ weights for each mouse, alongside uncropped images of the lungs of mice injected with each cell type, are available on figshare. DOI: https://doi.org/10.6084/m9.figshare.7633508.v1 ^12^.
