# Supplementary material for: Does the mouse tail vein injection method provide a good model of lung cancer?
Source: F1000Res. 2019 Feb 15;8:190. [Version 1] doi: 10.12688/f1000research.17964.1 (PMC6668049; doi:10.12688/f1000research.17964.1)
Supplement: Peer review history

# Peer review: Reviewer response for version 1

**DOI:** 10.5256/f1000research.19647.r50732

**Published:** 2019-07-29

**Authors:** Giulio Francia

**Affiliations:** 1 Border Biomedical Research Center, University of Texas at El Paso, El Paso, TX, USA

This is a neat paper describing tail vein injection to attempt to generate an *in vivo* model of lung cancer in mice.

 It would be helpful if the authors explain why they chose to use male SCID mice, and not female mice. They should also mention that PCR analysis of the lungs could be one way to look for potential metastases (by amplification of sequences that are human-specific).

 I think it would be helpful to include a side by side comparison (maybe a table?) of the tail vein results and the models mentioned in the intro, or results from other experiments that have established models using the cell lines mentioned in this study.

 They mention non-small cell lung cancer is the most prevalent subtype of lung cancer, but don’t discuss how the tail vein injection method could potentially model this specific type of lung cancer.

 The paper mentions intrathoracic implantation. Have the authors tried this method or are they merely stating it is an alternative option? Are the results comparable to the tail vein injection?

 They don’t state was size needle they use to inject in the tail vein.

 The authors could mention that a more laborious attempt (not without drawbacks) would be spontaneous metastasis assays, by first injection a primary tumor – followed by surgical resection – and then allowing several weeks for the development of spontaneous metastases.

I confirm that I have read this submission and believe that I have an appropriate level of expertise to confirm that it is of an acceptable scientific standard.

# Peer review: Reviewer response for version 1

**DOI:** 10.5256/f1000research.19647.r45833

**Published:** 2019-03-26

**Authors:** Malcolm Tingle

**Affiliations:** 1 Faculty of Medical and Health Sciences, University of Auckland, Auckland, New Zealand

The current study has attempted to use a relatively straightforward approach to determine whether injection of adenocarcinoma cells via a tail vein in mice can produce a viable model for lung cancer, presumably with a view to screening potential therapies for human disease. This appears as a reasonable aim, given that the current models are far from perfect, with the potential for a high failure rate of animals through technical difficulties with intrathoracic implantation. Although the current study is limited in its size and scope, it has produced results that are worthy of publication, in part because the model does not appear to have been as successful as perhaps the authors had initially hoped and that along the way there are clear welfare issues that should feature in any ethical harm: benefit consideration by an animal ethics committee.

 One of the key aims of this type of study is to mimic as closely as possible human disease. Unfortunately, the authors report that despite clear pathological changes occurring in the lungs of the animals, it was not possible to identify the presence of the cells of interest. Without further work-up, this would render the current strategy as potential for the screening of selective therapeutic approaches. The possibility for further workup may be limited by the fact that the tail injection of these adenocarcinoma cells appeared to produce lung damage that adversely impacted on the health of the animals, resulting in a decision to euthanise the animals. In this respect, the authors are to be congratulated on not merely adhering to some obvious metric, such as body weight, but also taking into account more subjective measures. One can only assume that ‘laboured breathing’, presumably as a consequence of the thromboembolism, was in fact quite marked, although the fact that animals did not suffer dramatic weight loss (Figure 1) indicates that they were still eating, and more importantly, not becoming dehydrated. To a large extent, any further attempts to develop this model will be limited by ethical consideration and oversight as science.

 The manuscript is succinct and given the findings of this preliminary study, the conclusions seem appropriate. However, I would recommend that the authors change the term ‘sacrifice”, since it really has no place in scientific literature, since it does not implicitly infer that the animal was killed humanely. The authors use the terms as if synonyms, “ *A weight loss of more than 20% was deemed to be unacceptable and would lead to early sacrifice of the mouse. At the end of 3 weeks in each experiment 4 mice from each group (i.e., 8 mice across the two repeats of the experiment) were euthanized by CO* *_2_ exposure*” However, there may be marked differences between the religious sacrifice of an animal and accepted species-specific practice of euthanasia for animals in scientific experiments covered by legislation.

I confirm that I have read this submission and believe that I have an appropriate level of expertise to confirm that it is of an acceptable scientific standard.
